# TNF-α and IL-8 Are Helpful in the Prediction of Late Post-COVID-19 Venous Thromboembolic Disease—Single-Center Prospective Study

**DOI:** 10.3390/ijms27209281

**Published:** 2026-10-19

**Authors:** Dorota Wyrostkiewicz, Katarzyna B. Lewandowska, Ewa Jankowska, Małgorzata Dybowska, Małgorzata E. Jędrych, Katarzyna Zimna, Adriana Roży, Monika Szturmowicz, Witold Tomkowski, Małgorzata Sobiecka

**Affiliations:** 1 First Department of Lung Diseases, Institute of Tuberculosis and Lung Diseases, Płocka 26, 01-138 Warsaw, Poland; k.lewandowska@igichp.edu.pl (K.B.L.); esrubka@gmail.com (E.J.); dybowska@mp.pl (M.D.); e.jedrych@igichp.edu.pl (M.E.J.); monika.szturmowicz@gmail.com (M.S.); m.sobiecka@igichp.edu.pl (M.S.); 2 Department of Genetics, Institute of Tuberculosis and Lung Diseases, Płocka 26, 01-138 Warsaw, Poland; adriana.rozy@gmail.com; 3 TS Clinic, Górczewska 228 E, 01-460 Warsaw, Poland; witotomkowski@gmail.com

**Keywords:** COVID-19, D-dimer, TNF-α, IL-8, cytokines, post-COVID syndrome, thromboembolism, venous thromboembolism

## Abstract

Post-COVID-19 syndrome is associated with persistent inflammation and an elevated risk of late venous thromboembolism (VTE). The role of proinflammatory cytokines has been well documented in the acute phase of the disease, but their potential role in late post-COVID-19 VTE remains unknown. What is the prevalence of VTE among symptomatic post-COVID-19 patients, and which laboratory parameters could enhance the diagnostic accuracy for VTE in these patients? Consecutive patients with objectively confirmed previous *SARS-CoV-2* infection, admitted to a single department of lung diseases in a tertiary hospital due to sustained symptoms of dyspnea and/or exercise intolerance, were included. Clinical assessment and laboratory testing, including cytokine profiling (Il-1β, IL-6, IL-8, IL-10, TNF-α), PAI-1, and ferritin, were performed. VTE events (pulmonary embolism and/or deep vein thrombosis) were confirmed by standard imaging. Clinical and laboratory parameters were compared between participants with and without VTE. 108 patients: 69 (63.9%) males and 39 (36.1%) females, entered the study. VTE episodes were diagnosed in 39.8% (16.7% in the course of COVID-19 and 23.1% after the acute phase of the disease). Patients who entered the study ≤6 months and >6 months from acute COVID-19 infection were compared. Median D-dimer concentration was higher in patients with VTE that occurred within 6 months from the COVID-19 diagnosis compared to the non-VTE group (607 ng/mL vs. 428 ng/mL, borderline significance, *p* = 0.0595). Median_IL-8 concentration was significantly higher in those who developed VTE more than 6 months after COVID-19 compared to non-VTE patients: 10.19 pg/mL vs. 6.61 pg/mL, respectively, *p* = 0.048. The same was noted for TNF-α (39.02 pg/mL vs. 24 pg/mL, respectively, *p* = 0.011). The best diagnostic utility for VTE prediction was obtained for TNF-α concentration in symptomatic patients diagnosed >6 months from acute COVID-19 infection (ROC AUC 0.73, 95% CI 0.57–0.91). TNF-α was an independent risk factor of VTE episodes in the whole study group. Detection of elevated IL-8 and TNF-α levels later than 6 months from acute COVID-19 disease may help to identify patients at increased risk of late venous thromboembolism; nevertheless, further studies are needed to validate this observation.

## 1. Introduction

Coronavirus disease 2019 (COVID-19) is a severe inflammatory illness caused by infection with *SARS-CoV-2* (severe acute respiratory syndrome coronavirus 2), which was first identified in China in December 2019 and later spread worldwide, becoming a pandemic in early 2020. It has resulted in over seven million deaths and nearly 800 million infections over four years. *SARS-CoV-2* infection triggers a generalized inflammatory response and can lead to severe organ function impairment, including the lungs, liver, kidneys, and intestines, as well as thromboembolic complications. The mechanism of thrombosis in COVID-19 is complex [1].

Hypercoagulability is a hallmark of severe COVID-19. *SARS-CoV-2* invades endothelial cells, causing damage and triggering a local inflammatory response that increases vascular permeability. Healthy endothelium has anticoagulant properties due to the regulated release of antiplatelet factors such as prostacyclin and nitric oxide. Additionally, endothelial surfaces help inactivating thrombin through antithrombin and convert it into an anticoagulant by interacting with thrombomodulin. Endothelial cells also produce tissue plasminogen activator (TPA), its inhibitor, and tissue factor pathway inhibitor (TFPI). *SARS-CoV-2*-induced endothelial damage impairs these anticoagulant mechanisms, fostering a prothrombotic state. Coagulation activation may also stem from reduced fibrinolysis and immune responses, which can trigger immunothrombosis [2].

Immunothrombosis is an effector pathway of innate immunity triggered by pathogens and damaged cells to limit pathogen spread and survival. While it may initially serve a protective role, uncontrolled immunothrombosis can lead to dysregulated coagulation and microthrombosis, or disseminated intravascular coagulation (DIC). In COVID-19, endothelial dysfunction and immune cell activation contribute significantly to the prothrombotic state. A key feature of immunothrombosis is the interaction between the hemostatic and immune systems, particularly involving monocytes, macrophages, and neutrophils. Proinflammatory cytokines, especially IL-6, promote platelet activation and aggregation. Neutrophil-derived serine protease cathepsin G also activates platelets [2].

*SARS-CoV-2*-induced endothelial apoptosis results in excessive expression of coagulation factors VII and VIII, as well as tissue factor (TF), and in the release of von Willebrand factor (vWF), thereby activating both the intrinsic and extrinsic coagulation pathways. Hypercoagulability is further promoted by disturbances in fibrinolysis, such as increased plasminogen activator inhibitor-1 (PAI-1) and decreased antithrombin activity. Persistent systemic endothelial activation may disrupt the structural and functional integrity of the endothelium. The endothelial surface is coated with glycocalyx—a glycoprotein-rich layer that regulates permeability, maintains microvascular perfusion, and prevents platelet adhesion by storing antithrombin and thrombomodulin [3].

In 2020, Iba et al. described these hemostatic changes as COVID-19-associated coagulopathy (CAC), characterized by elevated D-dimer and fibrinogen, minimal changes in clotting times, and predominant thrombotic over hemorrhagic complications. Early recognition of CAC, before DIC or VTE onset, is vital. Clinical characterization and risk assessment of COVID-19-related pulmonary embolism remain under investigation. Endothelial dysfunction appears pivotal in both acute and long-term complications [4].

Observations in COVID-19 patients show that hematological abnormalities underlying coagulopathy correlate with disease severity. Biomarkers such as fibrinogen, D-dimer, thrombin, and factors V and VIII aid in VTE risk stratification and prognosis, while inflammation markers (e.g., ferritin, IL-6) reflect endothelial injury. *SARS-CoV-2*-induced endothelial activation increases PAI-1, TF, and vWF expression, while thrombomodulin and tPA levels decline. Neutrophil-derived serine proteases, such as elastase, further promote prothrombotic states by inhibiting fibrinolysis. These changes elevate fibrin degradation products (D-dimers) and fibrinogen, reduce antithrombin levels, prolong PT and APTT, and elevate factor VIII. Fibrinogen measurement is crucial for assessing hemostasis, with D-dimer concentration being the most readily available marker of thrombotic risk and a poor COVID-19 prognosis [5].

The ISTH recommends measuring D-dimer, PT, platelet count, and fibrinogen in all COVID-19 patients. Cardiac troponin elevation in pulmonary embolism suggests myocardial injury, while elevated BNP and NT-proBNP indicate right ventricular strain. These markers should be assessed in all PE cases for risk stratification [6].

Pulmonary embolism and deep vein thrombosis—key manifestations of VTE—are frequent in both acute and post-COVID-19 phases, while arterial thrombosis (ATE) is less common. VTE incidence during acute *SARS-CoV-2* infection correlates with disease severity, ranging from low in mild cases to as high as 30 to 85% in critically ill patients. In 10–20% of severe cases, PE is a leading cause of death [7,8,9].

Accurate VTE prevalence in patients with COVID-19 is difficult to determine due to study heterogeneity. Early Chinese reports lacked PE documentation but noted elevated D-dimer and coagulation abnormalities. Subsequent studies confirmed PE cases globally, including in prospective trials. A review of 14 autopsy studies in 749 COVID-19 decedents found PE in 30%, with ICU VTE rates reaching up to 85.4% [10,11,12].

A 2020 meta-analysis of 86 studies (33,970 patients) estimated the overall VTE prevalence at 14.1%: 9.5% in non-screened patients and 40.3% in patients with ultrasound screening.

ICU patients had a VTE rate of 22.7% vs. 7.9% in general wards [13]. Another 2021 analysis of 18,000 hospitalized COVID-19 patients reported a 17% prevalence of VTE, rising to 25–33% with systematic screening or in critically ill ICU patients [14].

Considering all previous studies, we decided to evaluate the prevalence of VTE in symptomatic post-COVID 19 patients and to investigate the role of various biochemical and clinical factors in the prediction of post-COVID thromboembolic complications.

## 2. Results

One hundred and eight patients: 69 (63.9%) males and 39 (36.1%) females, mean age of 57.52 ± 13.4 years, entered the study. Mean BMI (Body Mass Index) was 30.57 ± 6.33. Fifty-seven participants (52.8%) were active or former smokers. Most of the patients had at least one comorbid condition, with arterial hypertension being the most prevalent (55 patients), followed by diabetes mellitus (DM) (20 patients), heart failure (17 patients), and ischemic heart disease (14 patients). Seven subjects reported a history of VTE in the past. Fifty-nine patients (54.6%) were hospitalized due to their COVID-19 symptoms, and the remaining 49 (45.4%) were treated at home. Among those hospitalized, 41 received nasal oxygen therapy, and 12 (11%) required high-flow nasal oxygen therapy (HFNOT). In four patients, mechanical ventilation was used (non-invasive in one; invasive in three—of which, one involved extracorporeal membrane oxygenation). The median time from acute COVID-19 to study enrollment was 150 days (range, 10 to 630 days).

Venous thromboembolic disease was diagnosed in 43 patients (39.8%). In 25 (23.1%) of them, VTE was recognized within 6 months of COVID-19, and in 18 (16.7%) cases, more than 6 months after COVID-19. Thirty patients (27.8%) were diagnosed with pulmonary embolism (PE), seven (6.5%) with DVT, and six (5.5%) with both PE and DVT. Ten patients were diagnosed with thrombosis in the proximal segment of the lower-extremity veins, two in the distal segment, and one in the internal jugular vein. Pulmonary embolism was observed in the lobar arteries in 9 patients, in the segmental arteries in 21, and in the subsegmental arteries in 6 cases.

DM and heart failure were diagnosed significantly more frequently in patients with VTE episodes; interstitial lung disease and coronary artery disease were significantly less frequent among those with VTE episodes (Table 1). No differences were observed between VTE and non-VTE patients concerning COVID-19 severity and treatment (Table 2).

Because of large differences in the time between COVID-19 and patients’ inclusion in the study, we analyzed laboratory parameters in two groups of patients: those who presented ≤6 months (group 1) and those who presented >6 months (group 2) from acute COVID-19 disease. The groups were comparable concerning laboratory parameters, except for aPTT and PT, which were prolonged in group 1 compared to group 2 (Table 3).

Subsequently, we compared VTE and non-VTE patients within the groups. In group 1, D-dimer concentration was higher in patients with VTE compared to non-VTE (borderline significance, *p* = 0.0595) (Table 4, Figure 1). In group 2, IL-8 and TNF-α concentrations were significantly higher in patients with VTE compared to non-VTE (*p* = 0.048 and 0.011, respectively) (Table 5, Figure 1).

ROC curves for VTE prediction based on TNF-α, IL-8, and DD concentrations were determined (Figure 2A–F and Table 6). The best diagnostic utility for predicting VTE episode was found for TNFα, in patients diagnosed >6 months from COVID-19 (AUC 0.73 95% CI 0.57–0.90).

Finally, we found the cut-off values for IL-8 and TNF-α that best discriminated patients with late onset VTE from those without VTE after COVID-19. They were 8.2 pg/mL for IL-8 and 26 pg/ml for TNF-α (sensitivity 76.5%, specificity 65.2%, positive predictive value—61.9%, negative predictive value—78.95%).

Multivariable logistic regression analysis, taking into account age, BMI, COVID-19 severity, coexisting DM, heart failure, or neoplastic disease in anamnesis, as well as TNFα and IL-8 concentration, confirmed an independent role of TNFα in VTE prediction. The other independent VTE predictor was BMI (Table 7).

## 3. Discussion

Venous thromboembolic disease is a common complication of COVID-19. It is known that *SARS-CoV-2* promotes thrombosis in both systemic and pulmonary vessels. VTE is usually reported in patients with severe COVID-19 who are treated in intensive care units (ICU), and it is rarely seen in those who are treated outside a hospital setting [15].

We present a group of patients with a history of *SARS-CoV-2* infection who were hospitalized due to persistent respiratory symptoms after recovering from COVID-19. The median time from the end of COVID-19 to study enrollment was 150 days (range, 10–630 days). Due to the observed differences in time from COVID-19 to the inclusion in the present study, we decided to divide patients into two groups: those diagnosed ≤6 months from acute COVID-19 (group 1) and those diagnosed >6 months from acute COVID-19 (group 2). Venous thromboembolic disease was diagnosed in 43 patients (39.8%). In 25 (23.1%) of them, VTE was recognized within 6 months of COVID-19, and in 18 (16.7%) cases, more than 6 months. To our knowledge, this is the first study focusing on both early and late VTE complications after COVID-19 illness. In most publications, the prevalence of VTE was assessed either during the acute phase of COVID-19 or shortly after recovery (60–90 days) [10,16]. This shows the need for active screening for VTE in COVID-19 patients with persistent respiratory symptoms.

We found a high prevalence of VTE in the presented group (39.8%). This exceeds the rates reported in other studies, which range from 14.7% to 27%, with higher rates exceeding 85% only in critically ill patients admitted to the ICU [8,9,14,17,18,19]. This difference may be due to selection bias. Patients in our cohort visited the hospital for ongoing symptoms, resulting in a higher proportion of affected individuals than in the general population of COVID-19 patients. Additionally, all patients, regardless of presenting symptoms, underwent lower-extremity venous ultrasound and CTPA. Studies that reported implementing screening procedures in asymptomatic patients (i.e., venous ultrasound of the lower extremities) revealed VTE prevalence as high as 40%, similar to our data [13].

More than half of our patients were hospitalized due to COVID-19, the majority receiving oxygen therapy during their hospital stay. However, only a few (4 subjects) were admitted to the ICU; the remainder were treated in the general ward. Severe COVID-19 was diagnosed in 51% of patients with VTE and 55% of those without VTE. On the other hand, as many as 49% of the patients with VTE were classified as having mild to moderate COVID-19, which indicates the need for VTE screening even in patients without respiratory failure due to COVID-19. The meta-analysis of Li et al. showed more pronounced differences in the COVID-19 severity between patients with and without thromboembolic complications, with 61% of those with VTE having severe disease [20].

We found that patients who developed VTE episodes were more frequently diagnosed with DM or heart failure than those who did not. Nevertheless, similar to other studies, we did not confirm their independent role in VTE prediction. [20,21].

In post-COVID patients, diagnosing VTE can be challenging due to prolonged symptoms and atypical presentations. D-dimer levels can be elevated at various stages of COVID-19, but this increase does not necessarily indicate VTE [12,22]. In our cohort, D-dimer concentration differed between VTE and non-VTE patients, but only in those diagnosed up to 6 months from acute COVID-19 illness; moreover, the median concentrations were not high—607 ng/mL and 428 ng/mL respectively—and only borderline significance was shown.

COVID-related coagulopathy is a complex condition involving multiple factors, including proinflammatory cytokines and proteins such as IL-1β, IL-6, IL-8, IL-10, TNF-α, ferritin, and PAI-1. Therefore, we evaluated these parameters as potential indicators of VTE likelihood. We found that in patients who entered the study >6 months after COVID-19, both IL-8 and TNF-α concentrations were significantly higher in VTE patients compared to non-VTE patients. The best diagnostic utility was shown for TNF-α, with AUC ROC 0.73 (95% CI 0.57–0.90). Optimal cut-off values were calculated from the ROC curves as 8.2 pg/mL for IL-8 and 26 pg/mL for TNF-α. This is the first report in the literature on the relationship between TNF-α and IL-8 concentrations and VTE diagnosed later than 6 months after acute COVID-19 disease.

Other studies have shown the strong proinflammatory activity of TNF-α, highlighting its role in promoting severe lung injury in patients with acute COVID-19 [23]. Furthermore, TNF-α, together with IL-10 and IL-23, was a predictor of a severe course of COVID-19 [24,25]. All of these studies were conducted during the acute phase of the disease. In one study by Siekacz et al., TNF-α concentration was elevated 3 months after COVID-19 in patients with persistent respiratory symptoms; however, its relationship to thromboembolic complications was not assessed [26]. A pre-pandemic 2018 study by Page et al. demonstrated the procoagulant effects of TNF-α and complement component C3 in vitro using thromboelastography and electron microscopy. TNF-α exposure promoted platelet aggregation and activation, suggesting that elevated C3 and TNF-α in inflammatory diseases may drive thrombogenesis [27].

Teodoro et al. [28] presented the concentrations of IL-1β, IL-2, IL-4, IL-6, IL-10, IL-17, IFN-γ, and TNF-α in patients with COVID-19 who developed thromboembolic complications in the acute phase of illness and found a significant increase in IL-1β, IL-2, and IL-10 levels in patients with thromboembolic events. TNF-α and IL-6 levels were also elevated in this group, but not significantly. It is noteworthy that the prevalence of thromboembolic episodes was low (7 cases in the group of 97 patients) and was assessed in acute COVID-19 [28].

A higher TNF-α concentration was reported by Alonso-Dominiquez et al. in patients with post-COVID syndrome, but it was significantly lower than that observed in our cohort (18.1 pg/mL) [29]. This finding supports our assumptions that the increased level of TNF-α in our study group was related to the thromboembolic complications, but not to the persistent post-COVID symptoms.

According to Li et al., serum levels of IL-8 correlated better than IL-6 levels with the overall clinical disease scores at different stages of COVID-19. Thus, IL-8 may act as a biomarker for COVID-19 disease prognosis and a target for COVID-19 treatment in the future [30]. Significantly elevated IL-8 concentrations were found in patients infected with the Delta variant of SARS-CoV-2 compared to those infected with Omicron, which correlated with more severe pulmonary inflammation and higher rates of hypoxemia in those infected with the Delta variant [31].

### Study Limitations

The main limitation of this study was its single-center design and relatively small patient cohort. However, most study groups cited in the discussion were of comparable size (50–97 patients). Moreover, our study had a prospective design; therefore, all of the patients underwent CTPA and venous ultrasonography of the lower extremities. This resulted in a higher diagnostic rate of VTE compared to those found by other authors, possibly representing the more realistic prevalence of VTE episodes in symptomatic patients post-COVID-19 disease.

The study showed significantly higher TNFα and IL-8 concentrations in patients with VTE episode compared to non-VTE patients, if diagnosed >6 months from COVID-19 disease. Whether this represents the pro-inflammatory profile responsible for late post-COVID thrombosis requires further validation in much larger group of patients.

## 4. Materials and Methods

### 4.1. Study Group

Consecutive patients who were admitted to a single department of lung diseases in a tertiary hospital due to sustained symptoms of dyspnea and/or exercise intolerance after *SARS-CoV-2* infection were included. All patients gave informed consent to participate in the study. The study was accepted by the Bioethical Committee of our institution on 10 December 2020. As a single-center study, it was not registered in any external database.

### 4.2. Study Protocol

All patients provided a detailed history of symptoms related to *SARS-CoV-2* infection, comorbidities, smoking status, treatments, and underwent a physical examination. All patients had objectively confirmed previous *SARS-CoV-2* infection (historical PCR or antigen test, or antibodies against *SARS-CoV-2*). Blood samples were collected for morphology with a differential white blood cell count, coagulogram (PT, aPTT, INR, D-dimer, fibrinogen), CRP, cytokine profile (Il-1β, IL-6, IL-8, IL-10, TNF-α), PAI-1, and ferritin levels. Peripheral blood from each patient was drawn during venipuncture and collected in a serum separator tube (4.9 mL). The serum tubes were allowed to clot for at least 30 min at room temperature (RT). The tubes were centrifuged (1000 rcf for 10 min at RT), and serum was aliquoted into 500 µL. All biological samples for cytokine measurement were frozen and stored at −80 °C for further experiments.

Serum concentrations of IL-1β, IL-6, IL-8, IL-10, and TNF-α were measured using the MILLIPLEX^®^ Human Cytokine/Chemokine/Growth Factor Panel A Magnetic Bead Panel 96-Well Plate Assay (HCYTA-60K; Merck Life Science, Darmstadt, Germany) on the Luminex MAGPIX^®^ system (Luminex, Austin, TX, USA), according to the manufacturer’s instructions. PAI-1 concentrations were measured using the FineTest^®^ Human SERPINE1 (Plasminogen Activator Inhibitor 1) ELISA Kit (FineTest, Wuhan, China), with absorbance measured using a Tecan Infinite M200 microplate reader (Tecan, Männedorf, Switzerland), according to the manufacturer’s instructions.

Patients underwent computed tomography pulmonary angiography (CTPA) using a Revolution Evo or Revolution GSI (GE Healthcare, Chicago, IL, USA). Compression ultrasound (CUS) examination of the lower limbs was performed using a GE Vivid S70N device with a linear transducer with a frequency range of 2.7–7.8 MHz. It consisted of assessing the proximal and distal deep venous system. The distal investigated vessels were the posterior fibular and fibulotibial veins of the gastrocnemius and soleus muscles. Diagnosis of VTE was established if venous ultrasound revealed deep venous thrombosis (DVT) of the lower extremities and/or CTPA revealed pulmonary embolism (PE), or both. Venous segments were examined in real-time B-mode in transverse sections. Lack of compressibility or direct identification of an endoluminal thrombus were the criteria used for the diagnosis of DVT. In one patient with a central venous catheter in the jugular vein, 2D ultrasound and color Doppler imaging revealed thrombosis in the common jugular vein around the vascular catheter. Ultrasound examinations were performed by experienced specialists in this technique (over 10 years of experience). They were blinded to the patients’ laboratory test results.

### 4.3. Statistical Analysis

Statistical analysis was conducted using GraphPad Prism 10.4.2 (534) (GraphPad Software, LCC, San Diego, CA, USA). For categorical variables (nominal and binary), percentage distributions were compared using the chi-squared test, or Fisher’s exact test in cases of small sample sizes. For continuous variables, the choice of statistical test depended on the data distribution within groups: variables with approximately normal distribution were analyzed using means (with standard deviation) and Student’s *t*-test, while those with non-normal distribution were assessed using medians (with interquartile ranges) and the Mann–Whitney U test. A *p*-value of <0.05 was considered indicative of statistical significance. Diagnostic performance of the analyzed parameters was assessed using receiver operating characteristic (ROC) curve analysis. The area under the ROC curve (AUC) was calculated to quantify discriminative ability of separate biomarkers.

## 5. Conclusions

Among patients with persistent respiratory symptoms after recovering from COVID-19, the prevalence of VTE was high—almost 40%. TNF-α and IL-8 were biochemical markers that differentiated patients with VTE from those without, in the group with late-onset VTE (more than 6 months after COVID-19 diagnosis). Moreover, TNFα was an independent predictor of VTE episodes in the whole study group. D-dimer concentration showed borderline significance in those who developed VTE in the first 6 months after COVID-19. Our research suggests that TNF-α and IL-8 could be used as additional parameters to assess the risk of late-onset VTE in post-COVID patients, especially considering the limited utility of D-dimer concentration in this population. Further prospective studies are needed to find out the role of both markers in other populations of patients at risk of developing VTE.

## Figures and Tables

**Figure 1:**
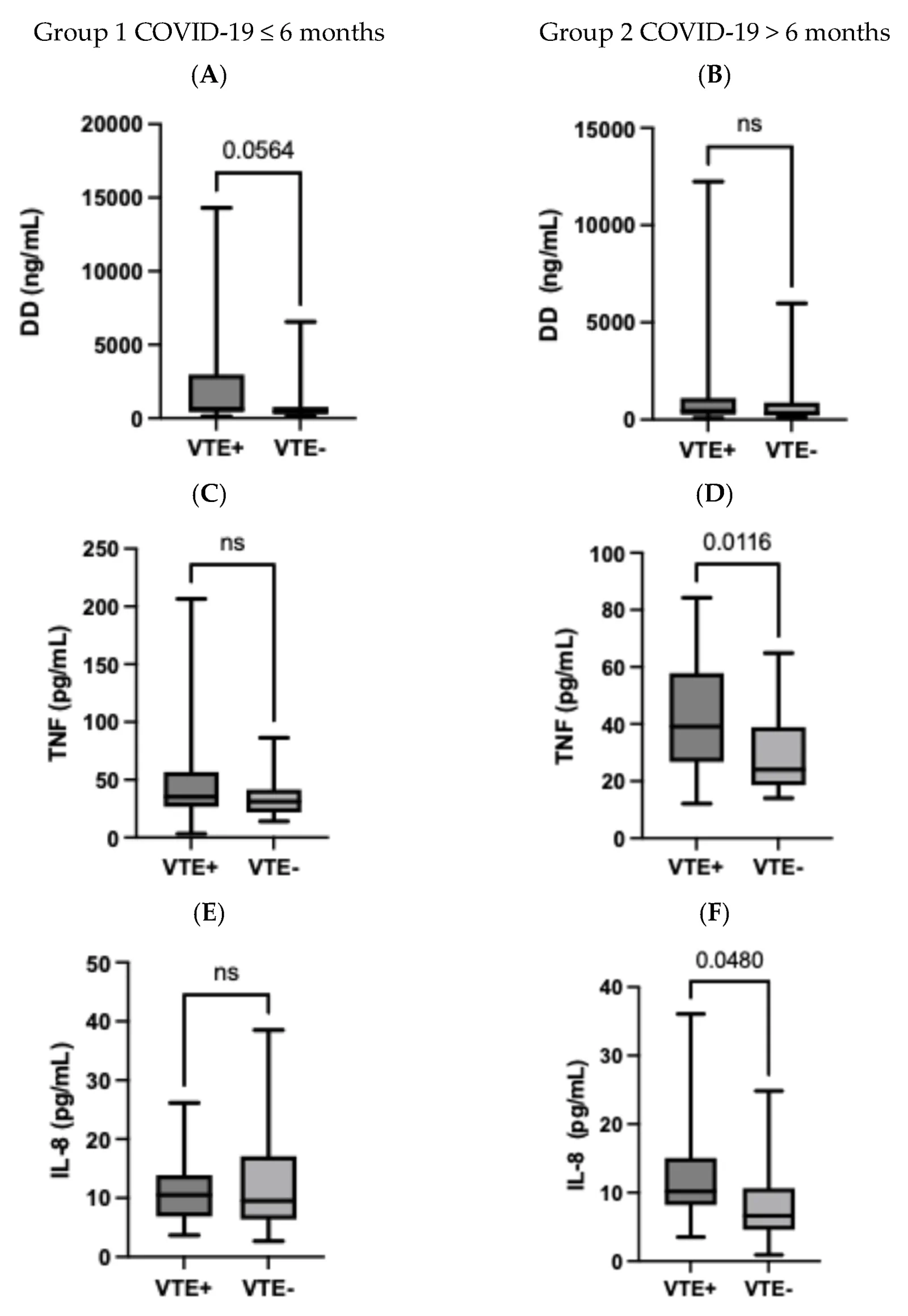
(**A**–**F**) Comparison of D-dimer (**A**,**B**), TNF-a (**C**,**D**), and IL-8 (**E**,**F**) concentrations in VTE(+) and VTE(−) patients, according to time from COVID-19 (group 1 ≤6 months from COVID-19, group 2 >6 months from COVID-19).

**Figure 2:**
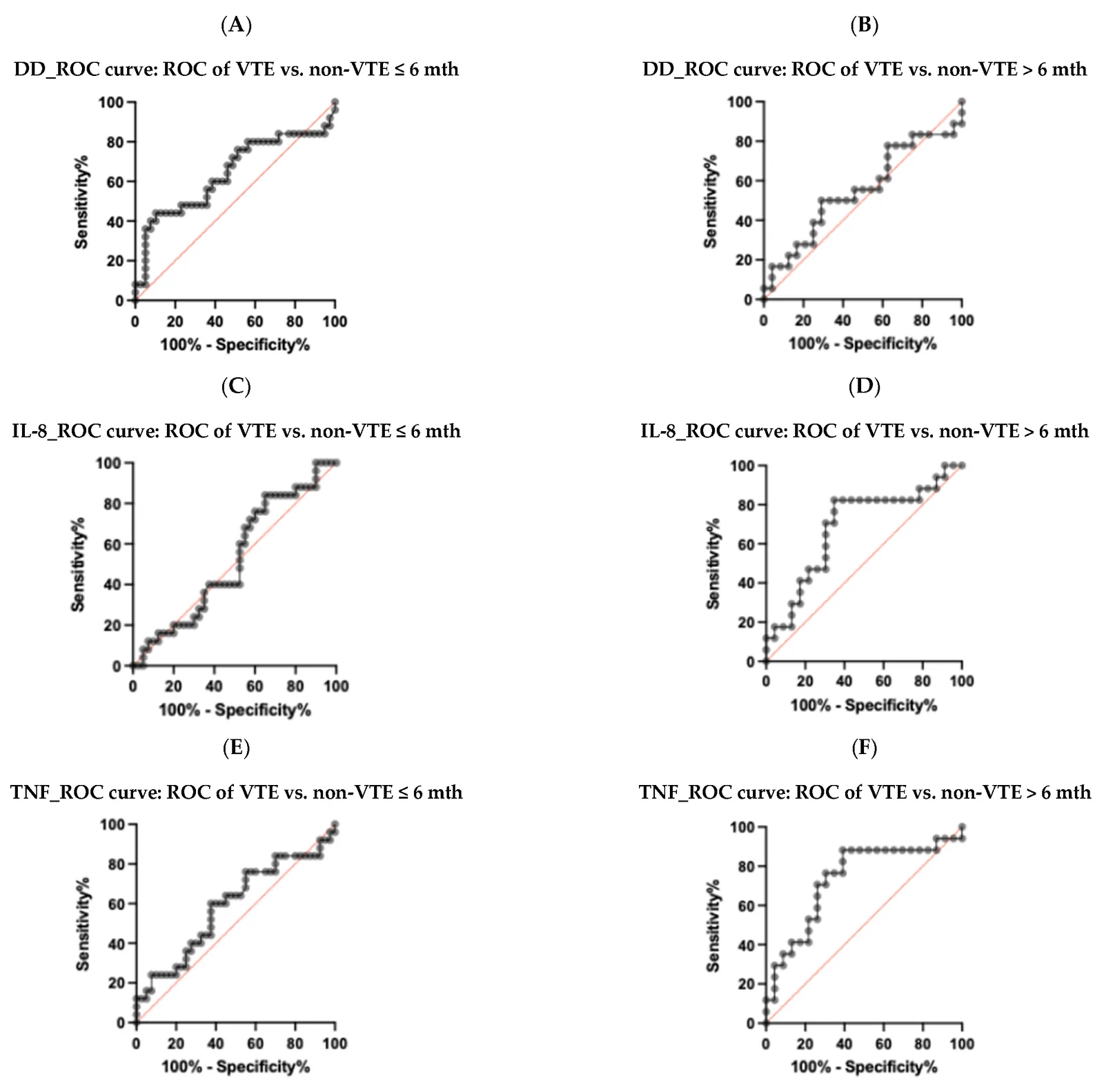
ROC curves illustrating the diagnostic utility of DD (**A**,**B**), IL-8 (**C**,**D**), and TNFα (**E**,**F**) for VTE prediction in patients included in the study ≤6 months and >6 months from COVID-19 diagnosis.

**Table 1:** Demographics and comorbidities.

DEMOGRAPHICS	VTE Nº43	Non-VTE Nº 65	*p*
Age—y (mean ± SD)	57.7 ± 13.04	57.4 ± 13.31	0.910
Sex—female—N (%)	17 (39.53)	22 (33.85)	0.661
BMI (mean ± SD) kg/m^2^	31.34 ± 6.54	29.54 ± 5.25	0.120
Smoking status—N (%)	19 (44.18)	38 (58.46)	0.930
pack-years ^a^ (mean ± SD)	18.38 ± 12.23	20.09 ± 16.62	0.720
**COMORBIDITIES**			
Arterial hypertension—N (%)	21 (48.84)	34 (52.31)	0.844
**Diabetes—N (%)**	**13 (30.23)**	**7 (10.77)**	**0.021**
**Heart failure—N (%)**	**13 (30.23)**	**4 (6.15)**	**0.001**
**Interstitial lung disease—N (%)**	**2 (4.65)**	**13 (20)**	**0.026**
**Coronary artery disease—N (%)**	**2 (4.65)**	**12 (18.46)**	**0.043**
Obstructive pulmonary disease—N (%)	5 (11.63)	14 (21.54)	0.209
Cardiac arrhythmia—N (%)	4 (9.30)	7 (10.77)	0.999
History of VTE—N (%)	4 (9.30)	3 (4.62)	0.433
Rheumatic diseases—N (%)	2 (4.65)	1 (1.54)	0.562
Neoplastic diseases—N (%)	2 (4.65)	4 (6.15)	0.999

VTE—recognized venous thromboembolism, non-VTE—without venous thromboembolism, SD—standard deviation, BMI—body mass index, N—number. ^a^ A pack-year is equal to smoking 1 pack (or about 20 cigarettes) per day for a year.

**Table 2:** COVID-19 severity and treatment.

	VTE Nº 43	Non-VTE Nº 65	*p*
COVID-19 stage I–II	21 (48.9)	29 (44.6)	0.67
COVID-19 stage III–IV	22 (51)	36 (55.4)	
**Treatment**			
Without oxygen—N (%)	21 (48.84)	29 (44.62)	0.291
Oxygen therapy (low-flow)—N (%)	13 (30.23)	28 (43.08)	
HFNOT—N (%)	7 (16.28)	5 (7.69)	
other: NIV, MV, ECMO—N (%)	2 (4.65)	3(4.62)	
Systemic CS—N (%)	17 (39.53)	32 (49.23)	0.466
Antibiotics—N (%)	30 (69.78)	39 (60)	0.311
LMWH—N (%)	20 (46.51)	27 (41.54)	0.690
Aspirin—N (%)	2 (4.65)	9 (13.85)	0.300
Remdesivir—N (%)	7 (16.28)	11 (16.92)	>0.990
Tocilizumab—N (%)	1 (2.33)	3 (4.62)	>0.990
Baricitinib—N (%)	3 (6.98)	4 (6.15)	>0.990

VTE—recognized venous thromboembolism, non-VTE—without venous thromboembolism, stage I—mild clinical symptoms, saturation >94%—no hospitalization, stage II—moderate clinical symptoms, saturation 90–94%, stage III—symptoms of pneumonia, saturation <90, oxygen therapy, HFNOT, or NIV required, stage IV—respiratory failure requiring mechanical ventilation support. HFNOT—high-flow nasal oxygen therapy, NIV—non-invasive ventilation. MV—mechanical ventilation, ECMO—extracorporeal membrane oxygenation support, CS—corticosteroids, LMWH—low-molecular-weight heparin.

**Table 3:** Laboratory parameters in two groups: group 1 ≤6 months, and group 2 >6 months after acute COVID-19.

Parameters:	Nº of Patients (Whole Group)	≤6 Months After COVID-19 N^o^ 66 Median (IQR)	>6 Months After COVID-19 N^o^ 42 Median (IQR)	*p*
Platelets [10^9^/L]	108	262.0 (208.8–325.3)	235 (197.5–272.3)	0.067
**APTT [s]**	**108**	**31.15 (28.48–35.0)**	**28.50 (26.18–31.5)**	**0.004**
**PT [s]**	**108**	**12.8 (11.78–14.13)**	**11.90 (11.08–12.90)**	**0.009**
D-dimer [ng/mL]	106	558.0 (300.8–1298)	352.0 (222–995)	0.069
Fibrinogen [g/L]	98	3.38 (2.56–4.35)	3.4 (2.92–3.99)	0.880
CRP [mg/L]	108	4.5 (1.48–14.35)	3.55 (1.48–10.6)	0.554
NT-pro-BNP [pg/mL]	98	70.49 (32.74–185.8)	153.4 (52.33–279)	0.090
Leukocytes [10^9^/L]	108	7.82 (6.41–9.63)	7.61 (6.44–11.11)	0.521
PAI-1 [ng/mL]	105	32.31 (26.71–40.01)	29.47 (25.87–33.68)	0.149
IL-6 [pg/mL]	104	2.49 (0.95–5.96)	2.12 (0.8–3.93)	0.294
IL-8 [pg/mL]	105	10.50 (6.50–16.13)	8.64 (5.57–12.72)	0.145
IL-10 [pg/mL]	105	1.78 (0.72–6.42)	1.615 (0.72–5.76)	0.436
TNF-α [pg/mL]	105	32.54 (23.46–42.46)	27.76 (22.06–44.16)	0.306
Ferritin [ng/mL]	105	101.6 (39.64–150.2)	85.87 (53.35–189.9)	0.949

**Table 4:** Blood parameters in patients with VTE vs. non-VTE (Group 1, ≤6 months from COVID-19).

Group 1	VTE N^o^ 25 Median (IQR)	Non-VTE N^o^ 41 Median (IQR)	*p*
Platelets [10^9^/L]	260 (182.5–323.0)	265 (212–329)	0.582
APTT [s]	32.2 (27.85–39)	30.90 (28.55–34.60)	0.634
PT [s]	13.2 (11.95–14.75)	12.6 (11.65–13.7)	0.195
INR	1.17 (1.07–1.32)	1.13 (1.03–1.225)	0.173
**D- dimer [ng/mL]**	**607 (413.5–2960)**	**428 (289–781)**	**0.0595**
Fibrinogen [g/L]	3.705 (2.828–4.548)	3.160 (2.48–4.2)	0.275
CRP [mg/L]	8.3 (1.4–21.2)	4.2 (1.5–9.3)	0.332
NT-pro-BNP [pg/mL]	52.43 (29.3–262)	87.68 (41.37–174.4)	0.538
Leukocytes [10^9^/L]	7.81 (6.51–10.31)	7.93 (5.78–9.49)	0.476
PAI-1 [ng/mL]	36.11 (26.97–41.53)	31.01 (26.40–38.22)	0.597
IL-6 [pg/mL]	2.46 (1.03–5.70)	2.56 (0.95–6.34)	0.907
IL-8 [pg/mL]	10.52 (6.84–13.9)	9.53 (6.35–17.09)	0.746
IL-10 [pg/mL]	2.57 (0.72–6.96)	1.53 (0.72–5.88)	0.422
TNF-α [pg/mL]	35.26 (26.56–56.86)	31.2 (21.74–41.55)	0.241
Ferritin [ng/mL]	108.6 (50.97–152.4)	92.14 (37.75–137.5)	0.582

**Table 5:** Blood parameters in patients with VTE vs. non-VTE (Group 2, >6 months from COVID-19).

Group 2	VTE N^o^ 18 Median (IQR)	non-VTE N^o^ 24 Median (IQR)	*p*
Platelets [10^9^/L]	228 (201.3–291.3)	239 (190.8–268.3)	0.836
APTT [s]	30.05 (27.38–34.93)	27.90 (25.3–30.15)	0.120
PT [s]	12.0 (11.4–13.6)	11.65 (10.78–12.78)	0.195
INR	1.07 (1.02–1.21)	1.035 (0.965–1.138)	0.190
D-dimer [ng/mL]	453.0 (251.3–1115)	340 (208.8–881)	0.584
Fibrinogen [g/L]	3.645 (3.068–4.328)	3.32 (2.895–3.955)	0.350
CRP [mg/L]	4.65 (1.7–12.5)	2.85 (1.13–5.58)	0.258
NT-pro-BNP [pg/mL]	177.8 (102.2–335.9)	84.76 (32.0–293.0)	0.0799
Leukocytes [10^9^/L]	8.575 (7.023–12.71)	7.315 (5.985–9.608)	0.127
PAI-1 [ng/mL]	29.82 (27.15–34.77)	28.65 (25.54–31.15)	0.221
IL-6 [pg/mL]	2.82 (1.45–7.01)	1.46 (0.565–3.268)	0.082
**IL-8 [pg/mL]**	**10.19 (8.2–15.06)**	**6.61 (4.57–10.66)**	**0.048**
IL-10 [pg/mL]	2.52 (0.98–6.32)	1.12 (0.72–3.31)	0.137
**TNF-α [pg/mL]**	**39.02 (26.62–57.78)**	**24.00 (18.52–38.95)**	**0.0116**
Ferritin [ng/mL]	73.09 (46.55–110.1)	87.8 (53.35–198.4)	0.692

**Table 6:** ROC curves for DD, IL-8, and TNF-α.

		AUC	Std Error	95% CI
DD	Group 1	0.64	0.076	0.49–0.79
	Group 2	0.55	0.093	0.37–0.73
IL-8	Group 1	0.52	0.073	0.38–0.67
	Group 2	0.68	0.088	0.51–0.86
TNF-α	Group 1	0.59	0.075	0.44–0.73
	Group 2	0.73	0.084	0.57–0.90

**Table 7:** Multivariable logistic regression analysis of the clinical signs and biochemical parameters predicting VTE development in patients after COVID-19 (whole study group).

Variable	Odds Ratio	95% CI	*p* Value
Age	1.008	0.974–1.044	0.665
**BMI**	**1.096**	**1.013–1.195**	**0.022**
COVID-19 severity	0.551	0.220–1.338	0.189
DM present	0.648	0.171–2.267	0.500
Neoplasm present	0.419	0.023–3.777	0.462
Heart failure present	2.349	0.606–10.58	0.222
**TNF-α**	**1.029**	**1.007–1.057**	**0.007**
IL-8	1.009	0.938–1.083	0.812

## Data Availability

The original contributions presented in this study are included in the article. Further inquiries can be directed to the corresponding author.

## Abbreviations

APTT	Activated partial thromboplastin time
BMI	Body mass index
BNP	Brain natriuretic peptide
COVID-19	Coronavirus disease 2019
CRP	C-reactive protein
CTPA	Computed tomography of pulmonary angiography
DIC	Disseminated intravascular coagulation
DVT	Deep vein thrombosis
ICU	Intensive care unit
IL	Interleukin
INF	Interferon
ISTH	International Society on Thrombosis and Hemostasis
NT-proBNP	N-terminal prohormone of brain natriuretic peptide
PE	Pulmonary embolism
PT	Prothrombin time
ROC	Receiver operating characteristic
SARS-CoV-2	Severe acute respiratory syndrome coronavirus 2
TF	Tissue factor
TNF-α	Tumor necrosis factor-alpha
TPA	Tissue plasminogen activator
TFPI	Tissue factor pathway inhibitor
vWF	von Willebrand factor
VTE	Venous thromboembolism
